# Three-Dimensional Imaging in Stem Cell-Based Researches

**DOI:** 10.3389/fvets.2021.657525

**Published:** 2021-04-14

**Authors:** Fariborz Nowzari, Huimei Wang, Arezoo Khoradmehr, Mandana Baghban, Neda Baghban, Alireza Arandian, Mahdi Muhaddesi, Iraj Nabipour, Mohammad I. Zibaii, Mostafa Najarasl, Payam Taheri, Hamid Latifi, Amin Tamadon

**Affiliations:** ^1^The Persian Gulf Marine Biotechnology Research Center, The Persian Gulf Biomedical Sciences Research Institute, Bushehr University of Medical Sciences, Bushehr, Iran; ^2^Department of Integrative Medicine and Neurobiology, School of Basic Medical Sciences, Institute of Acupuncture and Moxibustion, Fudan Institutes of Integrative Medicine, Fudan University, Shanghai, China; ^3^Department of Obstetrics and Gynecology, School of Medicine, Shiraz University of Medical Sciences, Shiraz, Iran; ^4^Laser and Plasma Research Institute, Shahid Beheshti University, Tehran, Iran; ^5^Department of Stem Cells and Developmental Biology, Cell Science Research Center, Royan Institute for Stem Cell Biology and Technology, Academic Center for Education, Culture and Research (ACECR), Tehran, Iran; ^6^Department of Physics, Shahid Beheshti University, Tehran, Iran

**Keywords:** stem cell, tissue clearing, three-dimensional imaging, mesenchym stem cell, microscope

## Abstract

Stem cells have an important role in regenerative therapies, developmental biology studies and drug screening. Basic and translational research in stem cell technology needs more detailed imaging techniques. The possibility of cell-based therapeutic strategies has been validated in the stem cell field over recent years, a more detailed characterization of the properties of stem cells is needed for connectomics of large assemblies and structural analyses of these cells. The aim of stem cell imaging is the characterization of differentiation state, cellular function, purity and cell location. Recent progress in stem cell imaging field has included ultrasound-based technique to study living stem cells and florescence microscopy-based technique to investigate stem cell three-dimensional (3D) structures. Here, we summarized the fundamental characteristics of stem cells via 3D imaging methods and also discussed the emerging literatures on 3D imaging in stem cell research and the applications of both classical 2D imaging techniques and 3D methods on stem cells biology.

## Introduction

Stem cells have greatly influenced our perspectives on mammalian development, disease and medical therapy. Animal and human studies have begun to identify the remarkable potential of stem cells for treating different diseases such as cancer (1), cardiac failure (2), diabetes mellitus (3), azoospermia (4), liver disease (5), ischemic stroke (6), Huntington's disease (HD) (7), and Parkinson's disease (PD) (8). They point toward the stem cells as a powerful source for cell replacement, with control of proliferative and self-renewing to sustain organic equipoise in the course of lifelong perturbations. Enormous advances have been made in assaying differentiation of a stem cell into a specific cell type combined with immunophenotypic, morphological, and functional criteria. It is now well-understood that stem cells are not a unitary type but contains three main types: embryonic stem cells (ESCs); tissue-specific (adult) stem cells including mesenchymal stromal/stem cells (MSCs), and induced pluripotent stem cells (iPSs).

Moreover, this field has garnered considerable progress supported by extensive histopathological analysis research using light microscopy, fluorescent probes and excised biopsies methods, providing the evidence for morphology-based and functionality-based principles in stem cell research. However, investigations have been recently started to identify the exact functions of the above stem cells in the biological system. Nor do we completely understand diversity of stem cells, which are involved in the disease modification and the architectural and cellular complexity of recurrent connections in particular organisms. An emerging theme is that connectivity patterns among stem cells have a main role in determination of diverse functionalities. Compared with traditional two-dimensional (2D) cell culture techniques, three-dimensional (3D) assays have been shown to better maintain gene expression, cell polarity, and cell contacts (9, 10). Furthermore, assessment and application of 3D imaging techniques on stem cells can be also brought under the spotlight. In addition, the development and application of tissue clearing methods brought new prospects of looking deeply into biological and therapeutic mechanisms of stem cells.

3D cellular medium might be thick and highly scattering which prevent deep penetration of light into the samples. The surface of the 3D medium could be easily monitored with common imaging techniques that work upon light reflection. However, 3D imaging techniques are utilized to image the whole outside and even inside of the 3D cellular medium. There are a large variety of optical imaging techniques to monitor 2D and 3D cellular systems (11). The 2D imaging techniques such as bright field and epifluorescence microscopies can be applied in serial-sectioning-based 3D imaging. Methods like stereo microscopy and differential interference contrast (DIC) microscopy have a great potential for creating a real-time 3D perspective of the cellular medium surfaces. There are a few methods [such as photo acoustic microscopy (PAM) and optical coherence tomography (OCT)] for 3D in-depth imaging of the cellular medium without any need of applying optical labeling agents. Furthermore, there are some powerful techniques for 3D imaging that can illustrate biological processes in a better resolution including: confocal, light-sheet, light-field, 2-photon and 4-pi microscopies. Meanwhile, the indispensable process before these imaging techniques is the optical clearing of the biological samples.

In the current review, we focused on some important findings related to the 3D imaging researches of stem cell niches architectures enabled by old (serial sectioning) and new (whole tissue clearing) technologies. Tissue clearing and 3D imaging methods reported during the current decade were reviewed with an emphasis on recent progress made with a novel and simple clearing method, fast free of acrylamide clearing tissue (FACT) technique, and its advantages were compared to the other whole tissue clearing methods.

## Whole Tissue 3D Imaging

### Serial Sectioning vs. Whole Tissue Clearing

The 3D imaging can be performed through two approaches. The first approach is based on serial sectioning and imaging using scanning microscopy. The tissues are cut into micron-diameter slices using the microtome. After imaging each sample, all images can be attached to reconstruct an intact 3D image from whole tissue using a computer software. Table 1 introduces software programs for 3D image reconstruction. Despite aiding in the expansion of science, this method has been dimmed due to problems and errors such as folding, pulling, tearing, and loss of some information, especially in cut-off areas of the texture and so on. Born was the earliest one who reported a 3D reconstruction of anatomical parts of amphibians based on serial sectioning using light microscopy (25) and after years, Denk and Horstmann (26) used block-face scanning or serial sectioning for acquired 3D image in electron microscopy. This kind of scanning can be performed in every microscopy to obtain 3D images. With this approach, each block of tissue can be imaged at a time as the layer of tissue is removed and destroyed after each imaging. Despite these limitations, this approach has been used recently to view different tissue textures (Table 2). In this method, a microtome was placed inside the chamber to make serial sectioning and imaging from each surface.

**Table 1:** Computer software programs for three-dimensional reconstruction of image series.

**Software**	**Company**	**Direction**	**References**
ImageJ	National Institute of Mental Health	http://imagej.net/Licensing	(12)
Imaris	Bitplane Corp	http://www.bitplane.com/	(13)
Mscope	PixeLINK Corp	ND	(14)
NI Lab view	National instruments Corp	http://www.ni.com/en-us.html	(15)
MetaMorph	Molecular Devices Corp	http://www.moleculardevices.com	(16)
Open lab, volocity	Perkinelmer Corp	http://www.perkinelmer.com	(17)
μManager	Ron Vale's laboratory	Open SPIM	(18)
Cell profiler	Carpenter Lab at the Broad Institute of Harvard and MIT	http://cellprofiler.org/	(19)
Neuronstudio	Computational neurobiology and imaging center	http://research.mssm.edu/cnic/tools-ns.html	(20)
VIAS	Computational neurobiology and imaging center	http://research.mssm.edu/cnic/tools-vias.html	(20)
L-measure	Developed by Ruggero Scorcioni	http://cng.gmu.edu:8080/Lm/	(21)
Huygens	Scientific Volume Imaging Corp	https://svi.nl/HuygensSoftware	(22)
SoftWoRx	DeltaVision Corp	ND	(23)
ZEN	Carl Zeiss	https://www.zeiss.com/microscopy/int/downloads/zen.html	(24)

*ND, no data*.

**Table 2:** Serial sectioning methods for three-dimensionally reconstruction of tissues image.

**Technique**	**Tissue**	**Microscopy method**	**Labeling method**	**References**
Array tomography	Nervous tissue	Two-photon microscopy, Scanning electron microscopy	Fluorescent labeling, heavy-metal staining	(27)
Plasma-mediated ablation	Nervous tissue	Two-photon laser scanning	Fluorescent labeling	(28)
Micro-optical sectioning tomography	Nervous tissue	Fluorescent microscopy	Fluorescent labeling	(29)
Serial two-photon tomography	Mouse brain	Two-photon microscopy	eGFP	(30)
Knife-edge scanning microscopy	Nervous tissue	White-light microscopy	Golgi-cox staining	(31)
Serial block-face scanning electron microscopy	Rodent brain, rats kidney, plants tissue, mouse pancreatic islet, *Trypanosoma brucei*, human retinal pigment epithelium	Scanning electron microscopy	Immune-gold labeling, heavy-metal staining	(26, 32–34)

After that, focus ion beam scanning electron microscopy (FIB-SEM) was used for 3D structural analysis. In this method, a focus beam of gallium ions was used to take a layer from the surface and a beam of electron was used to capture the surface. This technique can section a tissue into very thin layers (5 nm) to provide high resolution image of the tissue volume (35). Some examples of block-face imaging are mouse pancreatic islet (32), human retinal pigment epithelium (33), leaf cell and soybean root nodules (36) and single cell organism such as *Trypanosoma brucei* (37) (Table 2).

The second approach is based on whole tissue clearing (Table 3) followed by a 3D imaging technique (Table 4). The first advantage of this approach is the removal of slicing of tissue, which can then be used several times for imaging. However, the basic problem with this type of imaging of whole tissue is that since pictures are obtained from the entire texture with different thicknesses, it cannot provide a clear image of the deep parts of the tissue or dense tissue because of the light scattering or refraction. Some tissues have pigmentation creating the natural color of the tissue, and some of the tissues have fluorescent molecules creating an overlap with the fluorescent colors used for the creation of a specific image of a structure in the tissue (89). Another problem is the opaque tissue, which significantly restricts depth of imaging and thus creates low-quality images via light microscopy. Opaque backgrounds of the images of these tissues are because of an increase in the diffusion of light in them as well as distraction of light rays that move in a nearly straight path after colliding with a translucent tissue and tissue structures (cells and intracellular elements and extracellular matrix) (90). In addition, many studies on certain parts of the tissue, such as the study of the expression of genes, are not possible with most of these methods because the clearing process eliminates the structures of DNA and RNA in cells and tissues. Although, ribonucleotides labeling can be done using some methods of whole tissue imaging such as CLARITY (91).

**Table 3:** Different tissues clearing technique for three-dimensional imaging.

Technique^*^	**Tissue**	**Species**	**Microscopy method**	**Labeling method**	**References**
BABB (Murray's clear)	Lung, brain, kidney, liver, embryo, bone marrow, oocyte	Rodent, Drosophila, Xenopus	Electron ultra-microscopy, one-/two-photon confocal, light sheet	Immunofluorescence (DiI, DAPI)	(36, 38–47)
Spalteholz	Different kind of tissues	All animals	Just for macroscopic usage	NA	(48)
FocusClear	Different kind of tissues	All animals	Fluorescence, confocal	Immunolabelling, fluorescence labeling, lipophilic dye	(49)
ScaleA2	Brain, embryo, lung	Mouse	Electron ultra-microscopy, one-/two-photon confocal	Fluorescence labeling	(50, 51)
ScaleS	Different kind of tissues	Mouse, human	Electron ultra-microscopy, one-/two-photon confocal, light sheet	Immunochemical labeling, fluorescence labeling, lipophilic dye	(52, 53)
3DISCO	Brain, spinal cord, immune organs, tumor, lung, spleen, retinal organoid, mammary gland, lymph node, bone marrow cells	Mouse	Electron ultra-microscopy, One-/two-photon confocal, light sheet, wide-field epifluorescence	Fluorescence labeling, immunostaining (antibody staining)	(38, 40, 54, 55)
SeeDB	Brain, skeletal muscle	Mouse	Fluorescence, two-photon confocal	Fluorescent labeling, lipophilic dye (DiI, Sudan black)	(56–58)
Clear^T/T2^	Brain (embryo/adult), lymph node	Mouse	Confocal	Immune labeling, lipophilic labeling (DiI, CTB), fluorescence labeling	(59)
CLARITY	Different kind of tissues	Rodent, human	Two-photon confocal, light sheet	*In situ* hybridization, Immunohistochemistry, antibody labeling	(60–66)
iDISCO	Embryo, kidney, brain, pluripotent grafted cell	Mouse, human	One-/two-photon confocal, light sheet	Whole-mount immunolabelling (FoxP2, TrkA, TrkC, PAb #9,10), fluorescence labeling, nuclear labeling, immunocytochemical (EDU)	(67–69)
SWITCH	Brain	Mouse	Light sheet	Antibody labeling, fluorescence labeling	(70)
uDISCO	Different kind of tissues	Rodents	One-/two-photon confocal, light sheet, epifluorescence	Fluorescence labeling, immunolabelling	(71)
PACT/PARS	Different kind of tissues	Rodent, human	Confocal, scanning electron, fluorescence	*In situ* hybridization, immunohistochemistry, antibody labeling, fluorescence labeling, RNA probes	(63, 72)
CUBIC	Different kind of fresh fixed tissues, paraffin embedded of hospital archive samples	Rodent, primate, zebra fish, human	Fluorescence, single-photon confocal, light sheet	Fluorescent labeling, immunolabelling, nuclear labeling, Congo red staining	(73–77)
ACT-PRESTO	Different kind of tissues	All animals	Confocal, light sheet, fluorescence	Immunolabelling (centrifugal force or convection flow)	(24)
FastClear	Heart	Human	Two-photon confocal	Restricted in antibody labeling	(78)
FACT	Different kind of tissues	Rodent, bird	Confocal	Immunolabelling, genetic labeling	(79–81)

* *Methods are sorted according to the date of invention. NA, not applicable*.

**Table 4:** Review of 3D optical imaging method.

**3D imaging method**	**Advantages**	**Limitations**	**Range of depth**	**Range of resolution**
Serial sectioning	High lateral resolution	Invasive Soft tissues	Unlimited	0.5–100 μm mechanical sectioning (82) 0.5 μm lateral resolution of bright field microscope
Confocal microscopy	Ultra-high lateral resolution	High photo-toxicity	600 μm	Lateral resolution of 200 nm Axial resolution of 400 nm (83)
Light sheet microscopy	Short light exposure time, High penetration depth	Opaque compounds produces stripes	50 μm−5 mm (84)	Lateral resolution of 0.4–0.5 μm Axial resolution of 1.5 μm (85)
Light field microscopy	Single-shot 3D imaging	Low resolution Thick samples	60 μm (86)	1 μm (86)
Open SPIM	Experiment adjustable	Unpacked optical components	50 μm−5 mm (84)	Lateral resolution of 0.4–0.5 μm Axial resolution of 1.5 μm (85)
Two-photon microscopy	High penetration depth	High absorption in some agents (hemoglobin, melanin, etc.)	500 μm−1 mm (11)	Lateral resolution of 1.8 μm Axial resolution of 10 μm (87)
4-pi microscopy	Ultra-high axial resolution	Thick samples	9 μm (88)	Lateral resolution of 200–500 nm Axial resolution of up to 100 μm (11)

### Whole Cleared-Tissue Microscopy Methods

Stem cells can be seen in two forms of living and fixed cells by microscopy based on the goals of the study. Viewing live cells with an aid of microscopy makes it possible to study stem cell differentiation pathways through observing stem cell morphology. However, the imaging of fixed cells is required to investigate differentiated cells or examine the cells in the lineage in a population of differentiated cells, mature or immature, as well as to examine the structure of the tissue and cells and examination of the cells at any time of differentiation path. In addition, many stem cells cultured are opaque and thick in size and needed to be cleared and labeled for imaging with good resolution and structures (92).

There are some studies on 3D imaging of stem cells such as tumor stem cells, which are like to ESCs (93), with confocal microscopy, bright-field microscopy, fluorescence microscopy (for stained cells), phase contrast microscopy, light-sheet (almost use as investigate colonies development) (93, 94). There are several studies on stem cells with confocal microscopy such as research on isolation of human nervous system stem cells (95), visualization differentiation of stem cells to cardiomyocytes (96), colon stem cells (97), and hematopoietic stem cells (HSCs) (98, 99). However, in the following sections of the review, we explain the advantage of light sheet microscopy and its derivatives against confocal microscopy.

#### Confocal Microscopy

**Figure 1:**
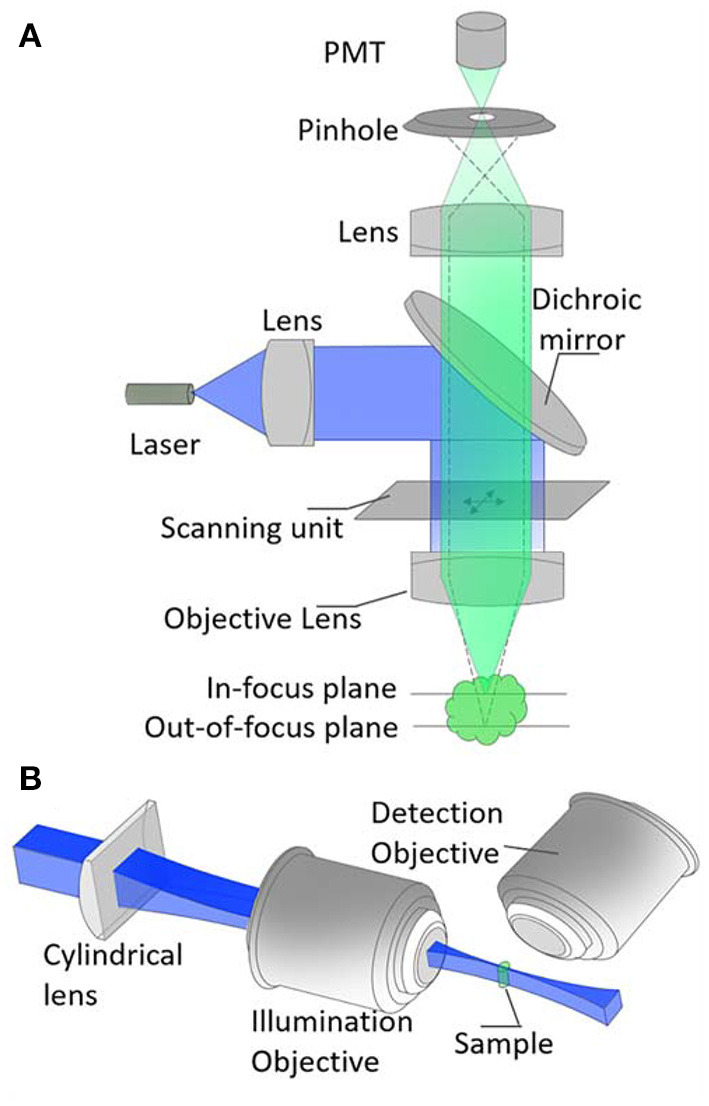
Optical setup of **(A)** confocal microscopy and, **(B)** light-sheet microscopy. The pinhole element in **(A)** helps to remove out-of-focus signals so that a high resolution image could be retrieved. The key element of **(B)** is the cylindrical lens. This helps to achieve a very thin sheet of light at the focus of the illumination objective. Blue and green represent excitation and fluorescence signals, respectively.

In the early 20th century, Richard Adolf Zsigmondy introduced the ultra-microscope. In this microscope, sunlight or white light passes through the gap and then passes through the condenser lens and becomes a focal point on the sample (Figure 1A). The sample is already presented in a colloid, and its image is formed with exposing it to light and reflecting the light from it (100). He was awarded the Nobel Prize in 1925 for his inventions (“Richard Zsigmondy” Nobel Laureates in Chemistry). Ninety years later, in 1993, this method was applied to fluorescence microscopes (101) and accordingly, a microscope called orthogonal-plane fluorescence optical sectioning (OPFOS) or tomography was introduced (101).

The problem in the resolution of the image captured by epi-fluorescent and other types of light microscopes attracted the attention of an American scientist named Marvin Minsky whose studies were in the field of cognitive sciences and artificial intelligence and he consequently invented the confocal microscope (102). This microscope sends a laser point to a part of the tissue and results in a focal image which solves the problem of lacking resolution in the previous microscopic method. In this microscope, out of focus light beams are removed by placing the pinhole on the focal plane and increasing the resolution and contrast of the image. As a few examples, Acar et al. utilized confocal microscopy for 3D imaging of stem cells in bone marrow (38), Zhang et al. used this microscope for 3D imaging of stem cell spheroids (103), and Haertinger et al. used it to analyze the 3D image of adipose stem cells (104). After overcoming the problem, valuable images have been taken using this technology, but there has been still a major problem relating to the image of thick tissues.

#### Light Sheet Microscopy

The light sheet microscope was most commonly applied for large specimens, and then the Selective/Simple Plane Illumination Microscopy (SPIM) was introduced by Huisken et al. (105). This method is capable of photographing live or fixed embryos and showing the expression of proteins, and is superior to previous methods (confocal), for example, there is no need for high levels of fluorophore to evoke and form the image, and also capable of taking image from deeper parts of a sample (105). Further investigations have shown that in this technique, damage due to light beams is much less (106, 107). Therefore, this method has quickly replaced previous methods for photographing embryos and studying their development.

The light sheet fluorescence microscope (LSFM) consists of two distinct optical paths, one for fast imaging of a wide range of samples, and the other one for imaging using a narrow sheet of light (Figure 1B). The light sheet is parallel to the focal plane of the detection path, and the thin layer of the light sheet is in the region where the sample is located. In many microscopes with one lens, there are two fundamental limitations: [1] Due to low numerical apertures, it is usually difficult to distinguish thin optical sections for a 3D image reconstruction, [2] Making a complete image from the whole sample with imaging from every micrometer of the sample increases the fluorophore bleaching and phototoxicity. In contrast, in SPIM, only the focal plane of the lens is selectively captured the whole sample's image that reduces its energy and prevents phototoxicity. In SPIM, each single light exposure of the sample (in about a few milliseconds) results in a fluorescence emission of the whole plane. The image of the plane is recorded using a wide field imaging system. The sample (or the light sheet) could be scanned to repeat the procedure for other planes of the sample. The plane-by-plane scanning provides a stack of 2D images to reconstruct a 3D image. However, in confocal microscopy, a single light spot only illuminates a tiny point on/in the sample and it requires a point-by-point scanning to reconstruct a 3D image.

Although the confocal microscopy has been considered as a powerful technique in high-resolution 2D imaging, the SPIM has some great potentials. First, the light exposure time to the sample is much less than confocal microscopy due to the plane-by-plane imaging mechanism. Second, loss of fluorescent signals in SPIM is very small. In comparison, the pinhole mechanism in confocal microscopy wastes some valuable image signals and a higher laser intensity should compensate the loss. Third, the SPIM is more powerful in depth imaging, while the confocal microscopy works upon tight light focusing and, due to scattering effects, it is difficult to have a perfect tight focusing inside the sample.

Thus, regarding this description, this method is a fast, safe and non-invasive method for examining fetal development and for examining tissues and all cases, which are sensitive to the photons (108). Furthermore, it can be used for large specimens, which require low-magnification and high-magnification lenses, such as large specimens (109). In this microscope, light rays collide as a light (thick or thin) sheet to the sample. These beams are created either by a cylindrical lens (105), or a normal laser focus up and down scanning technique, which allows us to use Airy or Bessel beams with more penetrating power and is used for thick sample (110–112). The Airy waveform was first expressed theoretically by Berry and Balasz (113), and then in 2007, the Airy beams were created and observed (114). These beams are used in microfluidic engineering and cell biology (115). Researchers at St. Andrews used these beams in a light sheet microscope (112). Their study showed that these beams increase the contrast of the image as well as the visual field by 10-fold. The better contrast is due to the asymmetric stimulation pattern of these beams that causes all of the stimulated fluorescents to contribute to a good contrast (112). In this microscope, the light rays are located vertically along the observation path. This light sheet hits the specimen, causing particles to be excited and propagated fluorescence radiation in the same thin layer as the beam is exposed. This fluorescence radiation is combined with a microscope object lens (perpendicular to the light plane) or processed by an imaging sensor (CCD, electron multiplying CCD, or CMOS camera) (108, 116). The Bessel beam, due to its non-diffracting nature, produces a very long and uniform sheet. Besides, the beam has a great advantage of self-healing. It means the beam can be partially re-formed after a tiny obstacle blocks its traveling path. The Bessel beam light-sheet was first reported in 2010 (117) and it is especially useful to image thick samples.

The sample is usually placed in a cylindrical container so that it can be seen from all directions of the sample without changing the sample position in the container. One of the most widely used microscopic environments is a low-melting agarose cylinder (106). Of course, as living specimens and growing embryos remain stable in this cylinder, they reduce growth and impairment of growth, to remove this problem, the sample in agarose is removed and moved to a cylindrical container containing the genus fluorinated ethylene propylene (FEP) with a refractive index (RI) of 1.34, which is near to RI of water, 1.33 (118).

According to the characteristics of this microscope, it is possible to insert and cultivate stem cells in a thin cylindrical tube to examine their distinction. Due to the benefits and good properties of this microscopy method such as multi-view imaging, less photo damage and high resolution and other mentioned above, this microscope have become a popular tool in biologic study especially in stem cells. Different designs of light sheet microscopy for different applications include light sheet-based fluorescence microscopy (106), line scanned light sheet microscope (110), digitally scanned laser light sheet fluorescence microscope (119), individual molecule localization-selection plane illumination microscopy (120), inverted SPIM (121), highly inclined laminated optical sheet (122), reflected light sheet microscopy (123), prism-coupled light sheet Bayesian microscopy (124), SPED light sheet microscopy (125), and lattice-light sheet microscopy (126). Here, we explain some studies on the use of light sheet microscopy in living or fixed stem cells and briefly explain their protocols.

Chen et al. (127) introduced a new light sheet microscopy, named lattice-light sheet microscopy and imaged ESCs. This microscopy method can be used to study large and more densely fluorescent tissues and specimens as well as a single cell in complex environments. This method can image at a high resolution, clarity and speed without photo bleaching (127). Another study on ESCs was conducted by Hu et al. (124) applied a novel design of light sheet microscope in which a prism was placed after the condenser objective (prism-coupled light sheet Bayesian microscopy). This design allowed them to use higher numerical aperture water immersion lenses and increased the field of view (124). In this study, images of both fixed and living human ESCs were obtained (124). In addition, there are many studies related to image of developing embryo of *Drosophila melanogaster* (128) and zebrafish (129). Another study was done on tibia bone marrow of mice for imaging HSCs with light sheet fluorescence microscopy (73). In this study, the bone marrow of tibia was cleared with ScaLeCUBIC-1 protocols and then imaged by ZEISS Z1 light sheet microscope (73). Furthermore, Allen et al. (130) injected stem cells intravenously to transgenic Zebrafish to investigate behavior of white blood cells via light sheet microscopy.

As above-mentioned in the protocols of light sheet microscopy, all techniques have some advantages and disadvantages, but the lattice light sheet microscopy has more potential for applying on stem cells studies due to fast imaging, low photo bleaching, and high resolution. Therefore, there is no major limitation in 3D imaging from all kinds of stem cells with this microscope as it is always improved and its limitations are becoming less than previous kinds of methods.

#### Light Field Microscopy

In 2006, Leovy et al. (131) at Stanford University developed a light-field microscope with changing optical microscopes. For the first time in 1908, Lippmann proposed the idea of placing a micro-lens array in intermediate image plane of a light-field camera. In the structure of the microscope, in the intermediate image plane, there is a micro-lens set. Each micro-lens transmits a part of the image onto the image sensor (usually Shack-Hartmann sensor) behind it. In fact, the micro-lens array helps to record the light filed information in a single shot. The light filed describes the magnitude and direction of light. The raw data are analyzed to reconstruct a 3D image (131). Until now, the microscope has been usually used to capture images of non-biological samples and fixed biological samples (131–133). Its advantage over other microscopes was that with a light exposure, a 3D image of the sample was quickly taken from the sample without the need to scan the entire sample volume. Another advantage of this method is the possibility of focusing on the image (132), which is very efficient for living specimens. One limitation of this method is the reduction of image spatial resolution by zooming on the picture that results in a significant reduction in the quality of image and this limitation has been improved using 3D deconvolution techniques (132, 134). Lu et al. (135) showed that high quality images can be obtained by combining the Shack-Hartmann sensor and a camera with high-resolution (CCD) and it is possible to zoom on the image without losing quality (High-resolution light-field microscopy).

Considering these facts, the light field microscopy has been also used for stem cell research. Choi et al. (136) used light-field microscopy for 2D imaging from kidneys after unilateral ureteral obstruction to investigate anti fibrotic effects of kidney-derived MSCs. Some studies have been conducted using this microscopy on stem cell-like cancer cells such as glioblastoma, renal cell carcinoma, and tumor-initiating stem-like cells and breast cancer (137–140), tumor induced in mice's by transplantation of human iPSs in dorsal flanks (141), hematopoietic cells in bone marrow (142, 143) and MSCs or its differentiated to skeletal myogenic injected in intra vertebra disc (144, 145).

#### OpenSPIM

The OpenSPIM whose name means the power to make changes in any research work, is a microscope, which uses a computer software on the Fiji open access platform (μManager), along with the open accessibility of microscopic hardware and provides researchers the ability to make changes to microscopic components in order to enhance the image quality of the microscope and does not require the use of a different types of microscope for different studies (18). In fact, this capability enables researchers, without expertise in optics, to make the specific microscope according to their own studies. On “https://openspim.org/,” you can share your microscope configuration or use others experience.

The first OpenSPIM microscope introduced has an illumination part and a detection part. Analysis of the image obtained by this microscope is done with the μManager program installed on the Fiji Oppenheim platform. The first specimen taken with this microscope was the zebrafish embryo, which expresses histone H2A-eGFP genes (18). This microscope showed the capability to capture rapid bioprocesses such as zebrafish fetal heart beats, and also to take images with a lot of light scattering. Images can be captured with multi-view and analyzed with μManager application (18). Rühland et al. (146) conducted OpenSPIM on fixed human hepatocellular carcinoma-cell spheroids that treat with human MSCs extracted from bone marrow.

#### Two-Photon Microscopy

Two-photon excitation is a non-linear optical process in which a biochemical probe absorbs two long wavelength photons (e.g., near infrared) simultaneously and emits a photon at the half wavelength of the absorbed ones (e.g., green). The mechanism could be applied in a two-photon microscopy. As the confocal microscope, the microscope is a point-by-point imaging system except for the light source and the pinhole. The two-photon excitation occurs only at high intensities. The light source is a pulsed laser and, high intensity condition is fulfilled exactly at the focal plane. Therefore, there is no need of the pinholes to remove background signals (11). Higher wavelengths provide longer penetration depths of 500 μm to 1 mm. The optical resolution of the microscope is better than OCT and PAM. However, due to the longer wavelengths, the optical resolution is lower than the confocal system. The two-photon microscopy could be useful to visualize collagen-enriched bone or tooth structures after hard tissue clearing (147, 148), 3D imaging of corneal stem cells in mouse eye (149), imaging of 3D stem cell spheroids (150) and even intracutaneous imaging of stem cell activity in a live mice (151).

#### 4-pi Microscopy

As dual-lens fluorescence microscopy, 4-pi microscopy is an axial super resolution technique that is especially useful in 3D imaging of sub-cellular structures. In a 4-pi microscope, the sample is illuminated from two opposite-direction lenses so that an interference pattern could be produced around the focus point. A principal bright fringe could be located at the focal plane and some weaker lobes. At the same time the dual lens collects the fluorescence signal from two opposite solid angles. Some mathematical post-processing removes the side lobes around the focal plane and an axially high resolution image could be reconstructed. The axial resolution could be improved 3–7-fold (11). The 4-pi microscopy could be utilized in ultra-high resolution 3D imaging of whole cell (88), 3D mitochondrial network of cells (152) and, even 3D structure of some endogenous nuclear proteins (153).

### Software Programs for Analyzing Cleared-Tissue Images

It is known that before the creation of these cameras, researchers were drawing microscopic images on paper or were later appearing them on the film, but today, image analysis software along with a digital camera, has made many improvements in the quality of microscopic images. The software synchronizes the structure of the microscope with the created image, correcting the flaws in the image created from each microscope and there is no need for difficult change in microscope components. So far, different software has been presented (Table 1).

## Transparency Methods: Advantages and Limitations for 3D Stem Cell Imaging

For 3D imaging of stem cells, there are some choices such as magnetic resonance imaging (MRI) or microscopy. MRI is the most commonly used method to evaluate the recovery process after the injection of MSCs into the organ of a live animal for example injection of MSCs in the myocardium of induced cardiac infarction pigs and observation of the therapeutic process (154). However, for more extensive studies with higher detail, microscopic methods have priority.

**Figure 2:**
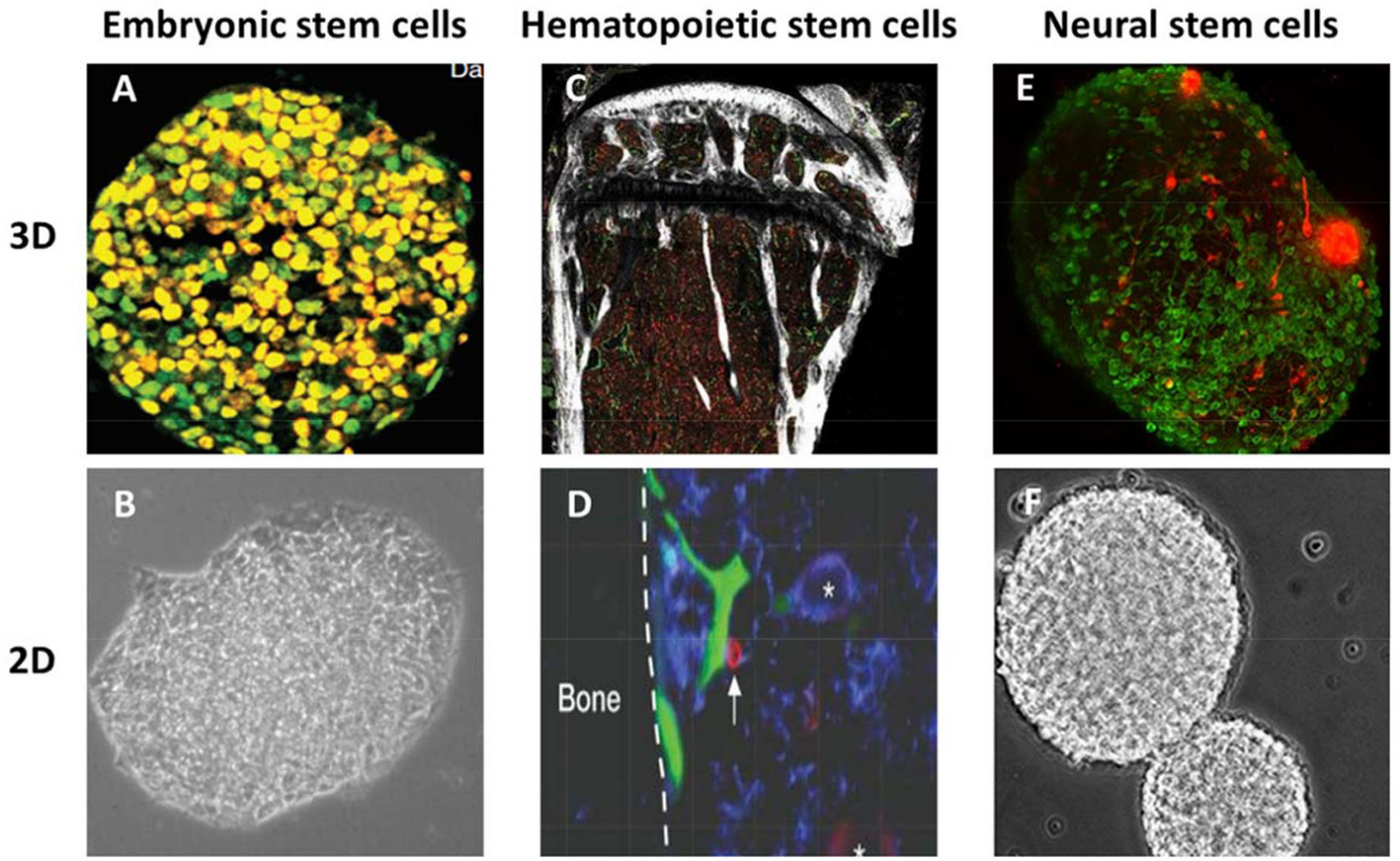
Three-dimensional (3D) and two-dimensional (2D) imaging of different types of stem cells. **(A)** embryonic stem cells after immunostaining and 3D imaging by laser confocal microscope (with permission) (155). **(B)** embryonic stem cell on day 1 by 2D imaging using phase-contrast microscope (with permission) (156). **(C)** 3D image of mouse femoral bone marrow cleared with CUBIC and imaged by confocal microscopy (with permission) (38). **(D)** 2D imaging of bone marrow hematopoietic stem cells by intravital microscopy (with permission) (157). **(E)** 3D imaging of neural sphere stem cells with light sheet microscopy (with permission) (158). **(F)** 2D imaging of neurospheres with phase-contrast microscopy (with permission) (159).

The tissue transparency methods for imaging stem cells can be divided into two groups in terms of their timing and progress: old classical methods for 2D imaging and then reconstructing the images to the 3D image (Table 2), and modern 3D imaging methods (Table 3). Using both 2D and 3D approaches, various types of stem cells have been imaged (Figure 2).

### Classical Methods for Tissue Clearing

From centuries ago, the samples were placed in a transparent and hard resin for clarity and preservation. Most of the resin materials used to hold the cleared specimens are water-proof and so the tissue water must be removed at the first step to keep them (160), but it should be noticed that resin embedding has some benefits for imaging, it protects clear tissue from fluorescence fading, mechanical damage and photo bleaching and provides long time for imaging sample without any unwanted change (160). The dehydration is usually done by several immersions in alcohol-water with increasing percentages of alcohol. After dehydration, the sample is put in a fat solvent. During this process, the membrane of the cell is also altered (161). Canada balsam is a type of resin creating a clear layer on tissues and is already clarified by xylene and then dehydrated. This resin itself has fluorescent properties, and is the best to use no fluorophore staining, because the background image become chromatic (161), which is not optimal in imaging and analyzing the image (24, 162). Resin embedding is useful in immobilizing cells for imaging, studying stem cells, and imaging scare cells sample by transmission electron microscopy (i.e., 10,000 cells) (143). In the early 20th century, Spalteholz introduced a clarification technique using organic solvents on a complete tissue (163). The result obtained was unprecedented at that time. This method requires several stages of dehydration, whitening and clarification, but it makes a great step in the field of anatomy and related sciences (163). One of the problems of this method is that during tissue preparation processes, about 1 cm around the tissue is dissolved and disappeared, so it is used only for large tissues (48). Despite of some disadvantages like performing just on anatomical sample or destroying some part of sample and longtime procedure, it has some advantage in anatomical science such as examining the cleared tissue after clearing and dissecting if necessary (48).

In the follow-up methods, for the sake of better 3D photography, fluorescence microscopes were most commonly used as imaging tools in the late 20th century. Before that, for microscopy and specific imaging of different parts of the tissue, it was required to produce a specific antibody to the specific structure in the tissue. These methods sometimes faced problem, especially in thick tissues with no antibody penetration at some depths of the tissue. After using new microscopy technologies, scientists were able to use fluorescent proteins, which then created images with very good specificity of structure in tissue and the problem of the previous method, namely, the inactivation of antibodies in some depths of the tissue, was solved (164). But in this method, there is also a problem of light dispersion, especially in thick tissues. This dispersion is due to the structure of tissue cells such as ribosomes, nuclei, nucleoli, mitochondria, membrane fat droplets, myelin, cellular skeletons and extracellular matrices such as collagen and elastin. Therefore, an effective and efficient clarification method can be used in today's research to eliminate or to some extent control these components, which cause the constraints and light dispersion.

For microscopy in *in-situ* conditions, it is first necessary to fix stem cells. For this purpose, after the culture of the MSCs isolated from tissue, they are placed in the hydrogel scaffold. After attaching to the scaffold, they can be used for fixation, clarification, labeling and microscopy. The epi-fluorescence microscope co-registered with optical coherence tomography has been used for imaging human MSCs (126). This combination has provided an efficient tool for investigating the behavior of these cells (126). In another study, Chen et al. (165) used the second near-infrared fluorescent imaging to find the recovery process of damaged skin in the intravenous injection of MSCs under *in-situ* conditions. They also used confocal microscopy for 3D imaging in *in-vivo* condition (165) and showed that intravenous injection slowly spreads to the injured area and accumulates at the margin of wound (165). For this purpose, several florescence dyes can be used for labeling MSCs such as indocyanine green (ICG) and lipophilic fluorescent dye such as DiD (166) or DiI (167).

Fluorescence microscopy is a good approach for tracing special parts such as MSCs in tissue by using immunofluorescence or expressing some genes for investigating protein expression and other application on biological research, but the most drawback is that fluorescence light is not permanent and it will fade in times by photo bleaching. In the following, we will express some protocols of clearing that influence in fluorescence keeping duration and their advantage and disadvantages. The most important features of fluorescence microscopy are its potential to improve causing to invent of many methods based on fluorescence and its imaging from living stem cells and regenerative medicine.

### Modern Techniques for Tissue Clearing and 3D Imaging of Stem Cells

**Figure 3:**
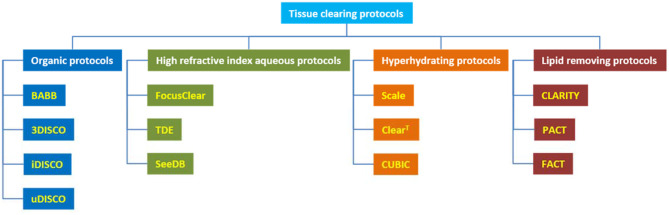
Tissue clearing protocols for three-dimensional imaging of stem cell in *in vitro* and *in vivo* conditions.

In fact, we need to minimize the difference RI between the sample and the environment. Today, the methods, which used whole tissue clearing, have been expanded. In general, four types of clearing have become widely used today (Figure 3): [1] methods based on Spalteholz method and organic solvents based methods, [2] high RI aqueous solutions based methods, [3] methods using hyperhydrating solutions based methods, and [4] lipid removal based methods. Advantages and disadvantages of various protocols have been summarized in Table 5.

**Table 5:** Advantage and disadvantage of whole tissue clearing methods in stem cell research.

**Technique**	**Advantages**	**Disadvantages**	**References**
BABB	1. Good transparency 2. Almost fast clearing (4 days)	1. Destroy fluorescence signal 2. Fixed sample only 3. Imperfect antibody penetration 4. Tissue shrinkage 5. No lipid preserve 6. Toxic 7. Complex with almost 32 steps	(168)
iDISCO	1. *In vivo* studies 2. Compatible for many labeling methods 3. Rapid 4. Better removal of lipid than 3disco	1. Tissue shrinkage 2. Fluorescence quenching after 2–4 days 3. No lipid preserve	(69, 169)
3DISCO	1. Enhance specific fluorescence signal due to reduction in background fluorescence 2. Efficient for lipid-rich tissues 3. Labeled structures remain intact 4. Good clearing without low change in tissue structure of retinal organoids	1. Fixed tissue only 2. Yellow fluorescent protein quench 3. Tissue shrinkage 4. No lipid preserve 5. Limited in immune staining 6. Cannot store in DBE for a long time due to structural destroy	(109, 170)
CLARITY	1. Protein preserving 2. Improve diffusion of probes in tissue due to removing lipid bilayers of the cell membrane 3. Less protein loss than other previous methods 4. Allow immunostaining	1. Morphological deformation and destruction 2. Transient swelling 3. Expensive 4. Time consuming (1–2 weeks)	(61, 171)
SWITCH	1. Multiple rounds of staining and destaining 2. Rapid	1. Toxic (sodium azide, glutaraldehyde) 2. Currently not used in many tissue (tick tissue, NA)	(40, 70)
ACT-PRESTO	1. Rapid (4–20 h for the whole organ) 2. Deep penetration of macromolecules 3. Allow all kind of immunostaining 4. Rapid immunostaining (3–4 h)	1. Expensive 2. Transient swelling	(24)
ScaleA2	1. Preserve fluorescent signals 2. Quantifying of the distance of different cells 3. Increase depth of confocal imaging 4. Inexpensive 5. Facilitate imaging with a water-immersion objective 6. High working distance	1. Led to tissue expansion 2. Long incubation time 3. Tissue fragile due to protein losses 4. Can't use for serial sectioning 5. Restricted to transgenic labels 6. More light scattering	(79, 168, 170, 171)
ScaleS	1. Preserve fluorescent signal and capable for immunostaining 2. No shrinkage 3. Tissue stable	1. Long process 2. Morphological deformation 3. Transient swelling 4. Restricted to transgenic labels 5. Need more time to month to clear high lipid and fatty tissue such as brain parts that have high myelin and muscle content	(52, 53, 79, 171)
CUBIC	1. Capable for various fluorescent labeling 2. Superior optical clearing 3. Non-toxic water-soluble chemical (easy to handle)	1. Morphological deformation 2. Transient swelling 3. Time consuming	(75, 171)
SeeDB	1. Lipophilic dye 2. Lipid preserve 3. No morphologic and chemical change 4. Inexpensive	1. Low resolution in light sheet microscopy 2. Restricted to transgenic labels due to not permeable to antibodies and macromolecules. 3. Brownish tissue but can be resolved by adding thiol 4. Low depth of light passing (1 mm)	(56, 57, 79)
ClearT	1. Maintaining the normal size 2. Lipid preserve 3. Increase depth of confocal imaging 4. Preserve fluorescence signals (when adding polyethylene glycol in clearing solution) but induce less transparency 5. Less time consuming then scale but with similar transparency	1. Can't use in agarose embedding 2. Restricted to transgenic labels 3. Little volume change	(59, 172)
PACT/PARS	1. Use for sparse elements (stem cells and metastatic tumor) in the whole body	1. Time-consuming 2. Transient swelling (remove in pars)	(173)
FASTClear	1. Ease and less time-consuming	1. Performed at 50°c and *in vivo* restricted 2. Restricted in antibody labeling	(78)
FACT	1. Simple 2. Most fluorescence preserving 3. Protein preserving 4. Rapid	1. SDS as a toxic chemical material	(79)

#### Organic Solvents

Several solvents have been used for the dehydration and clarification processes (174). The conventional dehydration solvent used in this method is methanol (with hexane or without hexane) or tetrahydrofurane (THF) (174). Both materials showed less effects on fluorescence proteins. Moreover, ethanol was used for this purpose, but this solvent may completely destroy green fluorescence protein (GFP). In addition, acetone, 2-butoxyethanol, dimethylformamide, dimethylsulfoxide and dioxan were used, but all of them had a negative effect on GFP (174). During the dehydration process, some of the fatty tissue dissolved. The removal of water and fat causes the formation of a high concentration of protein in the tissue. In fact, with the removal of other materials, the protein content of the tissue is increased, resulting in a higher RI of 1.5 more than the RI of fat and water. Consequently, in the process, solvents are needed to remove the remaining lipids from the tissue. So far, methylsalicilate, benzylalcohol, benzylbenzoate, dichloromethane, and dibenzylether have been used as final stage solvents (39, 48, 54, 67, 163, 174).

Good solvents must have several properties; first, they must have high fat dissolving capacity, secondly, their RI must be higher than 1.5, and thirdly, they must be affordable and safe. As mentioned, we need solvents with a high RI. Solvents with organic molecules have a high RI, especially those with a free electron, for example, solvents with a pi bond. Reagents with one or more aromatic rings have this feature and are suitable as the solvent. Fluorescent proteins require an aqueous medium for sustainability, but this method eliminates the stability of these proteins by tissue dehydration, and this is one of the limitations of this method.

##### Benzyl Alcohol–Benzyl Benzoate for 3D Imaging of Stem Cells

The BABB is one of the earliest clearing methods based on Spalteholz protocol (168). This method can be used just in small tissue and with high florescence expression because of its negative effects on fluorescence protein. After testing many materials, it was found that using THF instead of alcohol is better in protecting fluorescent protein (175). A study conducted by Acar et al. (38), modified BABB and 3DISCO were used for clearing bone marrow as investigating HSCs.

##### 3DISCO, iDISCO, and uDISCO for 3D Imaging of Stem Cells

Three methods based on tissue dehydration, 3DISCO (54, 175), iDISCO (67), and uDISCO methods (71), allow holding the fluorescent proteins in tissue for a few days. In 3DISCO, dibenzyl ether (DBE) is replaced with BABB in combination with THF. In addition, 3DISCO is used for clearing bone marrow cells of a transgenic mouse (38). However, this method alters the tissue structure specially membrane due to dissolving lipids and is useless for lipophilic dye, electron microscopy and living cells (175). Moreover, it cannot be applied to large tissues or whole body organs (71). Though, the use of this method on the retinal organoids has showed no problem in its structure and its shape, and it is suitable for clarification of this organ and imaging iPSs when combining with good immunostaining and confocal microscopy (40). The iDISCO has been used in numerous studies include Alzheimer's disease due to amyloidosis in mouse brain (68) and *in vivo* study of human pluripotent stem cells and can be used for monitoring graft biology and fate of cells (69). The uDISCO has been used to image transplanted stem cells within the entire body of adult mice (71).

#### High Refractive Index Aqueous Solutions

The disadvantages and limitations of solvent-based clarification have led scientists and researchers to work on methods that do not need to remove water from the tissue. All these water-based techniques, which have been built up, are based on one of these three solutions: [1] simple and inactive submersion of tissue in a solution with a RI near the tissue; [2] removal of tissue fats with tissue water supply (hyperhydration) to reduce the residual tissue RI; and [3] deletion of lipid active or inactive by immersion in an environment with equal RI with tissue.

The simple immersion is such that the specimen is immersed in a solution with a high RI. This method works well in small tissue, but if the texture is large, image quality is not as good as small specimens. In addition, in large tissues, a stronger intensity of clarity is needed. However, in any case, this method is an economical method because the main substance used for clarification is based on glycerol. The RI of glycerol is about 1.4–1.44. In this method, a tissue sample is put in a water-based solution containing a solvent (high RI) to inactive its clarification. Sucrose (28), fructose (56, 176), glycerol (177), 2,2- thiodiethanol (TDE) (178–180), and formamide (59) have been used for this purpose. These biological compounds called the RI match solution (RIMs) have been developed for establishing a proper RI (173). Diatrizoic acid (hypaque) used in FocusClear and Histodenz used in RIMs are complex molecules with an aromatic ring and three iodine atoms.

##### FocusClear for 3D Imaging of Stem Cells

FocusClear, a simple water-soluble reagent, was invented by Chiang (49) and is used for clearing biological animal/plant/organism tissues. Moreover, its use in 3D imaging of stem cell in mice intestine was reported by FocusClear (181).

##### TDE for 3D Imaging of Stem Cells

TDE, which is a clear-to-yellow, water-soluble and low-viscous liquid, is compatible with immunostaining and can be used for adjusting proper RI in the range of 1.3–1.515 by dissolving certain values. For the first time, TDE was used as a mounting media for tissue clarification for super-resolution microscopy (178). It can also clarify large tissues (176, 179) and is suitability used for confocal due to its feather of suppressing photo-oxidation. Moreover, it has a good tissue clearing ability and is suitable for two photon microscopy (182). One of the problems with TDE is that at high concentrations, the brightness of a number of fluorophores is reduced (178). TDE was used for 3D localization of stem cell in mice gut (183).

##### SeeDB for 3D Imaging of Stem Cells

SeeDB is a simple immersion water-based optical clearing agent using fructose and thiol that was firstly used on mouse brain for imaging dorsal to the ventral side of it (56). SeeDB contains a saturated solution of fructose in water for reaching its RI to 1.50 (in 37°C) and α-thioglycerol for inhibiting Millard reaction. SeeDB can clear the sample in 3 days and is compatible to many fluorescence proteins and as it is detergent-free, tissues and cellular structures like membrane were preserved and allowed to use lipophilic dye and tracers (56). The light sheet microscopy was not recommended for imaging SeeDB clearing samples due to low resolution (57). 3D human IPs spheres was imaged with SeeDB (184). This protocol was used for 3D imaging of enteroids *in vitro* (185, 186). 3D imaging of colorectal epithelial stem cells in mice was also done by SeeDB (187). For localization of neural stem cells after transplantation and differentiation in mice brain, SeeDB was used (188). Mammary stem cells was imaged in mice mammary duct by this method (74).

#### Hyperhydrating Solutions

In many cases, tissue enlargement is one of the side effects of this clarification, and it is known that as tissue grows, the duration of imaging of the entire tissue is increased. Furthermore, in some tissues, tissue texture and size need to be kept unchanged so that we can gain the correct information. Excessive tissue enlargement is prevented with increasing glycerol concentration, but as the higher concentration of glycerol results in less clarifying accrue, there is a limitation on increasing the concentration of glycerol/water. In addition, the effect of urea on proteins can lead to the loss of some tissue deformation (56).

##### Scale for 3D Imaging of Stem Cells

In the scale method, the detergent used for removing lipid contains a hydrating agent such as urea and glycerol (50). Hyperhydration reduces the RI to 1.38 (50). This method shows better preserving fluorescence, better clearing and better manipulation than CUBIC, 3DISCO, SeeDB (52). This method has been used in several studies include hippocampus neural stem cell spheres (168). This study showed using Scale and Clear^T2^ protocols on this tissue can enhance the depth of confocal imaging to 100 μm and also these protocols can be used for quantifying and measuring their geometric proportion and studying vascular niche in a neural stem cell in the sub-granular zone of dentate gyrus of a transgenic mouse (50). In another study, an adult mouse lung was used for clearing and imaging epithelial cells of type 2 to clarify the potential of maintenance and repair of these cells in tissue damages (51). Human MSCs in the zebrafish embryo were imaged using Sca*l*eU2 method (189). Sca*l*eA2 was used for imaging of colorectal epithelial stem cells in mice (187).

##### Clear^*T*^ for 3D Imaging of Stem Cells

Clear^T^ is an easy immersion method in which urea-like molecules are used for clarification. This method can also be applied to thick tissues (59). ClearT2 showed better performance in imaging neural cells and spheres than Scale and SeeDB (168). Due to its potential for maintaining the size of spheres and compatibility with typical immunostaining technique, its RI can also be changed by changing in component concentration that allows it to be used in various neural cell types (168).

##### CUBIC for 3D Imaging of Stem Cells

The CUBIC method based on urea combine with aminalcohols is one of the methods of hyperhydration with series of immersion steps followed by a washing step (75, 190). In this method, sucrose is used in a clarifying solution to increase the RI (75, 190). It also uses high-percentage Triton (50%) to increase the power of eliminating fat in tissue. CUBIC was used in the study of mammary glands and their differentiation as well as cells fate in mice mammary gland ducts (74). The CUBIC protocol was used to investigate skin stem cells in full thickness skin biopsies (191). HSCs were also imaged in the spleen, brain and thymocytes after clearing them cleared with CUBIC method (192).

#### Lipid Removal Methods

The novelty of lipid removal methods is using hydrogel (usually acrylamide) that acts as a barrier to prevent removal of DNA/RNA and protein structure during clearing with or without electrophoresis (60). In lipid removal protocols such as CLARITY and PACT/PARS (60, 61, 173), after embedding tissue in a hydrogel, the hydrogel is fixed, and the lipid is removed by incubation in a detergent such as sodium dodecyl sulfate (SDS) or by electrophoresis to accelerate the process. The final stage of clarification is performed using FocusClear or the ones previously described such as RIMs, TDE, 80% glycerol, histodenze and diatrizoic acid (70, 173, 193). Two other methods based on simple immersion that introduce in late 2016 and 2017, were Fast of Acrylamide SDS-based Tissue Clearing (FASTClear) and FACT that are free-acrylamide but worked such as previous lipid removal protocols (79, 194).

##### CLARITY for 3D Imaging of Stem Cells

CLARITY can be done on the whole organ and provides 3D image of structural and molecular from the tissue. Moreover, it was used for bone clearing and provided good results with access imaging up to 1.5 mm in mice femur and tibia (195). It was also used on bone osteoprogenitor cells and for imaging stem cells present in bone marrow (195). Using this protocol, tumor stem cells were traced in mice brain (196). Furthermore, mid-brain organoids were cleared and imaged using this protocol (197).

##### PACT for 3D Imaging of Stem Cells

Woo et al. (72) presented a modified-PACT (mPACT) with a better transparency and less proceeding time than PARS-mPACT for clearing whole CNS in mouse as well as rats and guinea pigs. In addition, PACT-deCal was introduced for bone clarification that can be used to enhance bone clarity and imaging adult stem cells within, and expansion-enhanced PACT (ePACT) was also introduced that can be used to examine cells and subcellular components with good magnification (198). By combining RIMs with PACT, PACT can provide good images in different tissues and can be applied to study stem cells and their relationship with the surrounding environment.

##### FACT for 3D Imaging of Stem Cells

**Figure 4:**
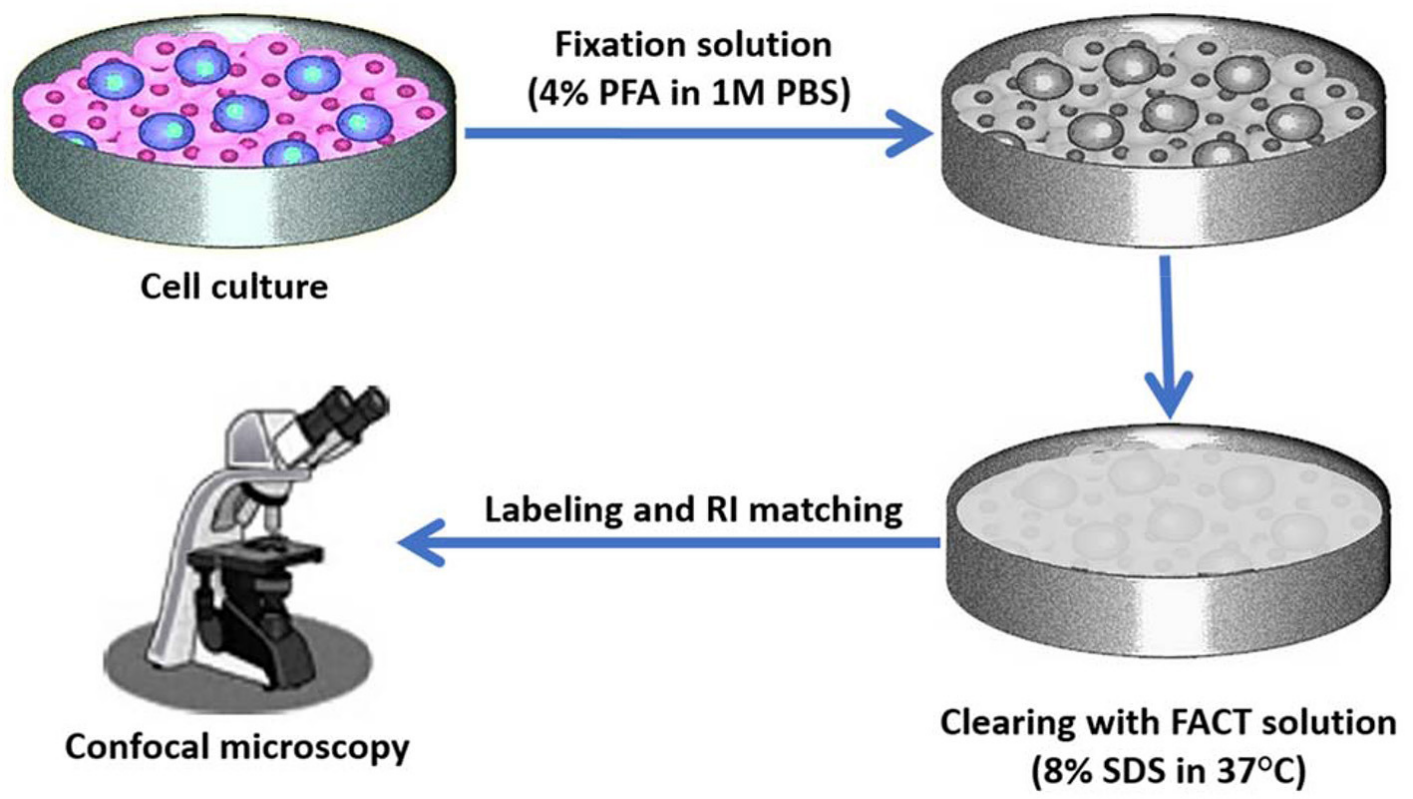
Cell culture three-dimensional (3D) imaging with the FACT method. Target labeled cells will be cultured in a traditional culture medium. After confluency of cells, they can be fixed in 4% paraformaldehyde (PFA) in phosphate buffer saline (PBS). After cell fixation, clearing FACT solution containing 4% SDS can be added and dishes can be shaken at 37°C. The cells then can be washed with PBS and after refractive index (RI) matching, 3D imaging can be performed by confocal microscope.

**Figure 5:**
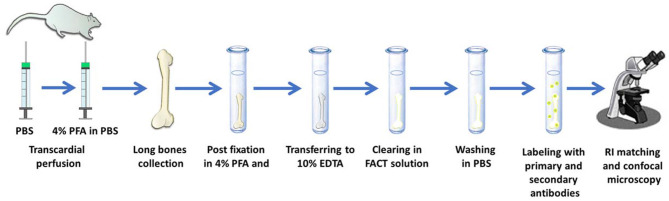
Locating bone marrow mesenchymal stem cells (BM-MSCs) by whole intact bone three-dimensional (3D) imaging based on the FACT method. Using 40 mL ice-cold phosphate buffer saline (PBS), transcardial perfusion of rats can be done and followed by 20 mL of 4% paraformaldehyde (PFA) in PBS. Then, collected long bones can be post fixed 3 days in 4% PFA solution. Then for demineralization of the bones can be incubated in 10% EDTA in 4°C. EDTA should be changed daily. Then, the bones can be cleared by the FACT solution (SDS 8% in PBS, pH 7.5). After clearing process and washing of SDS, the tissue can be labeled with specific antibodies for BM-MSCs and then after refractive index (RI) matching can be imaged by confocal microscope.

Xu et al. (79) compared the FACT clarification method with CLARITY, PACT, and FASTClear methods. Weight change was higher in methods with a hydrogel stage. High temperatures, inappropriate pH and immersion presented in previous methods caused inappropriate changes in the tissue, such as excessive tissue volume or massive weight changes, or some loss of information. FACT has been used for labeling tumor stem cells in the mice brain (199). The FACT protocol as a fast and cheap approach can also be used for 2D and 3D cultures of stem cells (Figures 4, 5).

### Selection of the Best Clearing Protocol for 3D Imaging of Stem Cells

Here, we described all clearing protocols in this article and explained the advantages and disadvantages of all methods. Most of these methods have been designed to do on the nervous system and a few of them have been reported to use on stem cells. It seems that FACT has the most potential among all of the previous methods in applying to stem cells because of less time requirement, proper temperature and proper pH, which are all essential factors for this purpose. Future studies are required for approving this capability. Finally, for choosing a proper method for clearing, some tips should be considered such as the purpose of the study and the type of tissue. If the sample is very large (as much as an adult mouse), there is a need for methods with the ability to make the whole organ transparent, such as CUBIC, CLARITY, PACT, uDISCO, and FACT. Of course, the speed of operation in large textures is also important, in which case, the FACT method is appropriate.

## Conclusions

The stem cell field is a multilayered and multidisciplinary challenge, the combination of tissue clearing methods with 3D imaging techniques has extended the previous work based on conventional 2D imaging techniques, and has paved the way toward a systemic study of stem cell systems and pathologies that underlie it. Modern methods of tissue clearing have potential for many studies and are the right direction of developing in clearing methods. Moreover, these methods combined with new microscopy methods such as LSFM and OpenSPIM with image analysis software can be applied to many studies especially on stem cell studies, such as stem cell tracking for regenerative medicine. The future trend toward a combination of both tissue clearing methods and 3D imaging can exploit the feasible application of stem cell systems both on physiology and disease. It is our hope that understanding the cell interactions and systematic characterization of stem cell systems may provide guidance for the development of new treatment strategies for disease-modifying therapies and regeneration of tissue defects.

## Author Contributions

AT, HL, IN, and MZ conceived, designed the format of the manuscript, and reviewed the manuscript. FN, HW, AK, MB, NB, AA, MM, MN, and PT drafted and edited the manuscript. All authors contributed to the critical reading, discussion of the manuscript, read, and agreed to the published version of the manuscript.

## Conflict of Interest

The authors declare that the research was conducted in the absence of any commercial or financial relationships that could be construed as a potential conflict of interest.
